# ColorPhAST: a visual rapid colorimetric assay for detecting phage-susceptibility in *Escherichia coli*

**DOI:** 10.3389/fmicb.2025.1638204

**Published:** 2025-07-11

**Authors:** Paula Gómez Estévez, Ángel Rodríguez-Villodres, Lydia Gálvez-Benitez, Guillermo Martín Gutiérrez, José Miguel Cisneros, José Manuel Ortiz de la Rosa, José Antonio Lepe

**Affiliations:** ^1^Clinical Unit of Infectious Diseases, Microbiology and Parasitology, University Hospital Virgen del Rocío, Seville, Spain; ^2^Institute of Biomedicine of Seville (IBiS), University Hospital Virgen del Rocío/CSIC/University of Seville, Seville, Spain; ^3^Centro de Investigación Biomédica en Red de Enfermedades Infecciosas (CIBERINFEC), Madrid, Spain; ^4^Department of Health Sciences, Loyola Andalucía University, Sevilla, Spain; ^5^Department of Medicine, Faculty of Medicine, University of Seville, Seville, Spain; ^6^Department of Microbiology, Faculty of Medicine, University of Seville, Seville, Spain

**Keywords:** bacteriophages, *Escherichia coli*, visual detection, rapid susceptibility test, new method

## Abstract

The alarming increase in the prevalence of multidrug-resistant microorganisms, which in some cases has left no available treatment options, has led to a renewed interest in previously abandoned therapeutic approaches such as bacteriophage therapy. Therefore, the development of rapid methods for determining phage susceptibility will be essential in the near future because phage-based treatments could provide a viable alternative for patients with infections caused by multidrug-resistant microorganisms. In response to this need, a new test named ColorPhAST (Color Phage Activity Susceptibility Test) based on color change of red phenol has been designed to detect phage-susceptibility in just 2 h. A total of 100 *Escherichia coli* isolates were used to evaluate the performance of the test, 55 being resistant to the isolated phage through the double agar overlay spot assay (gold standard method). The sensitivity and specificity of the test were 95.6 and 100%, respectively. The ColorPhAST is a fast, easy-to-performed, and accurate method with great potential for identifying susceptibility to bacteriophages.

## 1 Introduction

Phage therapy began to be widely used to treat bacterial infections in humans and animals in the 1930s. However, when penicillin entered the market in the early 1940s, phage therapy lost its importance due to the broad-spectrum activity of penicillin and future antibiotics against bacterial infections. In recent years, phage therapy has undergone a revival due to the growing problem of multidrug-resistant bacteria and the lack of resources for antibiotic research (Strathdee et al., 2023). In Russia and Georgia, phage cocktails are accessible without a prescription for treating bacterial infections of multiple etiologies (Gordillo Altamirano and Barr, 2019; Yang et al., 2023). By contrast, in Europe, phage therapy is regulated for use only in extreme cases, where it is provided as a compassionate treatment alongside antibiotic therapy. This limited application is due to the absence of specific regulations for its widespread clinical use (Gordillo Altamirano and Barr, 2019; Yang et al., 2023).

A phage’s host range refers to the range of bacteria it can infect. Some phages have a broad range, targeting multiple species within or across genera, while others are highly specific, infecting only one species or a few isolates (Strathdee et al., 2023). This specificity is an advantage as phage targets only harmful bacteria without affecting the microbiome, but it also requires a precise match between the phage and bacterium for effective therapy (Gordillo Altamirano and Barr, 2019). Therefore, before initiating treatment with bacteriophages, it is crucial to accurately assess the bacteria’s susceptibility to the phage.

Several phage susceptibility tests have been described for evaluating phage lytic activity. Plaque-based assays, including double agar overlay of phage-bacteria, double agar overlay spot, and cross-streak phage assays, being the oldest and most widely used methods. Other techniques for detecting phage activity include liquid media tests (LMT), which are based on growth kinetic assay, such as optical density assay or the OmniLog system (Yerushalmy et al., 2023).

Although several techniques for testing phage susceptibility have been developed, none so far effectively balances price, speed, accuracy, and ease of execution. In this study, we have developed a rapid (2-h) and cost-effective test for detecting phage-susceptibility in *Escherichia coli*, named Color Phage Activity Susceptibility Test (ColorPhAST), which might be used worldwide, regardless of the technical level of the laboratory.

## 2 Materials and methods

### 2.1 Bacterial isolates

A total of 100 isolates of *E. coli*, collected from clinical samples from the Microbiology Service at the University Hospital Virgen del Rocío (Seville, Spain) were used in the development and evaluation of the test. Identification of the isolates was performed using matrix-assisted laser desorption ionization–time of flight (MALDI-TOF) (Bruker, Germany). This collection included 55 phage-resistant *E. coli* clinical isolates and 45 phage-susceptible *E. coli* clinical isolates. A reference strain (*E. coli* ATCC 25922) was also used for phage isolation and titer determination.

### 2.2 Phage isolation, purification and preservation

For the isolation of phages, 1 mL of wastewater from the University Hospital Virgen del Rocío (Seville, Spain) was mixed with 5 mL of bacterial culture (*E. coli* ATCC 25922) in Tryptone-Yeast (TY) medium (Yeast extract 3 g/L, tryptone 5 g/L, CaCl₂ 0.7 g/L) in a Falcon tube (50 mL). It was then incubated for 48 h at 37°C with shaking. Following incubation, it was centrifuged at 8000 rpm for 15 min and the supernatant was filtered with 0.22 μm to eliminate bacterial cells.

For the purification, serial dilutions (10^−1^ to 10^−5^) of the phage filterate were prepared. For the double agar overlay of phage-bacteria, 100 μL of dilution 10^−3^ and 10^−5^ were added together with 200 μL of the bacteria and incubated for 15 min at 37°C. Then, the phage-bacteria suspension was added to the TY overlay medium (Yeast extract 3 g/L, tryptone 5 g/L, CaCl₂ 0.7 g/L, 0.65% agar) and poured into TY agar plates (Yeast extract 3 g/L, tryptone 5 g/L, CaCl₂ 0.7 g/L, 2% agar). The plates were incubated for 24 h at 37°C. Then, a clear lysis plaque was isolated with a sterile stick and was grown with 5 mL of TY medium (Yeast extract 3 g/L, tryptone 5 g/L, CaCl₂ 0.7 g/L) and 500 μL of bacteria (*E. coli* ATCC 25922) (1.5 × 10^8^ CFU/mL) at 37°C with shaking for several days. This solution was centrifuged at 8000 rpm for 15 min and the supernatant was filtered (0.22 μm). To purify and concentrate the phage lysate, polyethylene glycol 8,000 (PEG 8000) was added to the filtered supernatant to a final concentration of 10% (w/v), along with 1 M NaCl. The mixture was incubated overnight at 4°C, followed by centrifugation at 10000 rpm for 15 min. The resulting phage pellet was resuspended in SM buffer (100 mM NaCl, 30 mM MgSO₄H₂O, 50 mM Tris–HCl, 0.01% gelatine) at a pH of 7.5 to ensure stability. To eliminate residual PEG, 50 μL of chloroform were added, mixed, and centrifuged at 3000 rpm for 10 min, after which the supernatant was recovered. The isolated and purified phages were preserved at 4°C.

### 2.3 Determination of phage titer

To calculate the number of viral particles in the phage suspension, serial dilutions (10^−1^ to 10^−8^) were prepared. In Eppendorf tubes, 100 μL of 10^−6^, 10^−7^, and 10^−8^ dilutions were mixed with 200 μL of *E. coli* (ATCC 25922). Then, 300 μL of the mixture (bacteria + phage dilution) was mixed with 3 mL of TY overlay medium and poured into TY agar plates. A bacterial control without phage was also performed. The plates were incubated for 24 h at 37°C. Finally, clear lysis plaques were counted and plaque-forming units per millilitre (PFU/mL) was calculated.

### 2.4 Double agar overlay spot assay

In order to compare the results of the ColorPhAST with the results of a gold standard technique, the lytic activity of the phage was determined through the double agar overlay spot assay. Briefly, bacterial colonies were resuspended in sterile NaCl (0.85%) to achieve a 3 McFarland standard optical density (9 × 10^8^ CFU/mL). 200 μL of the bacterial suspension was mixed with 4 mL of TY overlay medium and poured onto TY agar plates. After the overlay solidified, 10 μL of phage suspension was spotted onto the surface and allowed to dry. The plates were incubated at 37°C for 24 h, and lysis was assessed by the presence of clear lysis plaques, indicating susceptibility to the phage.

### 2.5 Reagents and solutions

**Figure 1:**
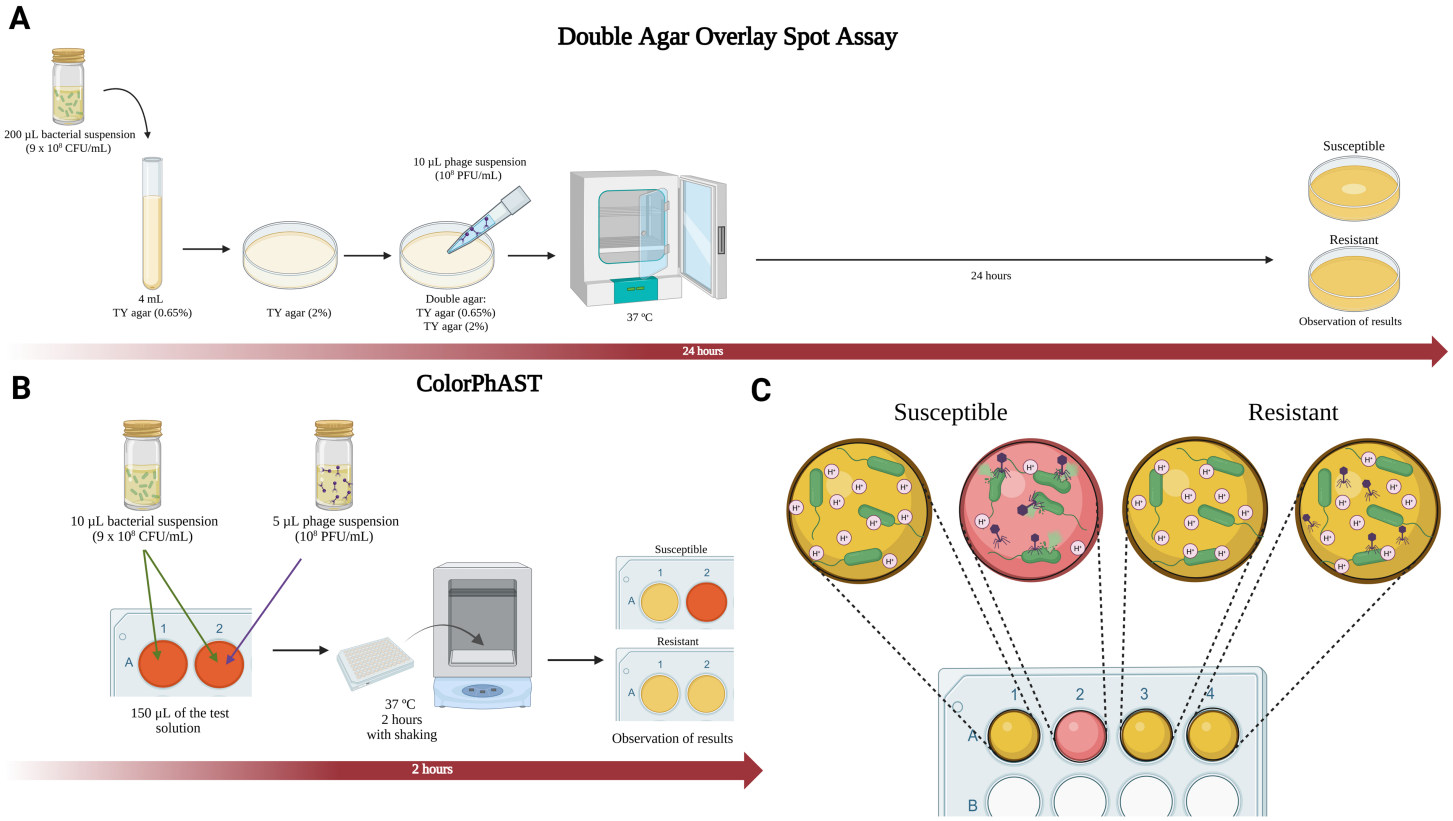
**(A)** The gold standard double agar overlay spot assay. **(B)** ColorPhAST. **(C)** Schematic diagram with interpretation of ColorPhAST results. *Figures created with Biorender.*

Following a similar experimental approach as previously described (Ortiz de la Rosa et al., 2023), ColorPhAST is based on a color change caused by the acidification of the phenol red solution, as a consequence of glucose metabolism carried out by the bacteria. The bacteria take up glucose from the test solution and metabolize it, resulting in the production of protons (H^+^), which decrease the pH and acidify the surrounding medium (Fuller and Kim, 2021) (Figure 1C). Briefly, the rapid test requires three reagents: Mueller-Hinton broth (MHB), D-(+)-glucose monohydrate and 0.5% phenol red solution (Sigma-Aldrich, Germany). The solution was prepared by mixing 47 mL of MHB, 700 μL of 0.5% phenol red solution and 2.5 mL of D-(+)-glucose (10%). The pH was further adjusted to 7.5, and after autoclaving the Mueller-Hinton broth (MHB) medium, sterile-filtered glucose (10%) and commercially sterile phenol red solution (P0290) were added to obtain a final concentration of 2.5% MHB, 0.007% phenol red solution and 0.5% D-(+)-glucose. This solution can be kept at 4°C for 1 week or at −80°C for 6 months and must be prewarmed at 37°C before use to prevent growth delay and a delayed color change.

### 2.6 ColorPhAST

Bacterial colonies were resuspended in sterile NaCl (0.85%) to obtain a 3 McFarland. Unlike RapidTZP (Ortiz de la Rosa et al., 2023), bacterial concentrations used was higher than ones used in RapidTZP (0.5–1 McFarland) trying to reduce the time to obtain the results. For each *E. coli* isolate tested, two wells of the plate were used. Both contained 10 μL of the bacterial suspension (3 McFarland) and 150 μL of the test solution. In the second well, 5 μL of the phage suspension (10^8^ PFU/mL) were also added. Regarding the incubation time, the absorbance was measured for three phage-susceptible and three phage-resistant strains at different incubation times: 30, 60, 90, 120, 150, and 180 min (data not shown). It was determined that the earliest incubation time allowing for a clear distinction between sensitive and resistant strains was 120 min (2 h). Thus, the inoculated wells were incubated at 37°C with shaking for 2 h. After the incubation time, both wells of each strain were observed. If both wells have undergone a color change from orange to yellow, it means that the bacteria have grown both in the absence and in the presence of the phage. In this case, the phage is not capable of lysing the bacterial strain, which means that we are dealing with a phage-resistant isolate.

**Figure 2:**
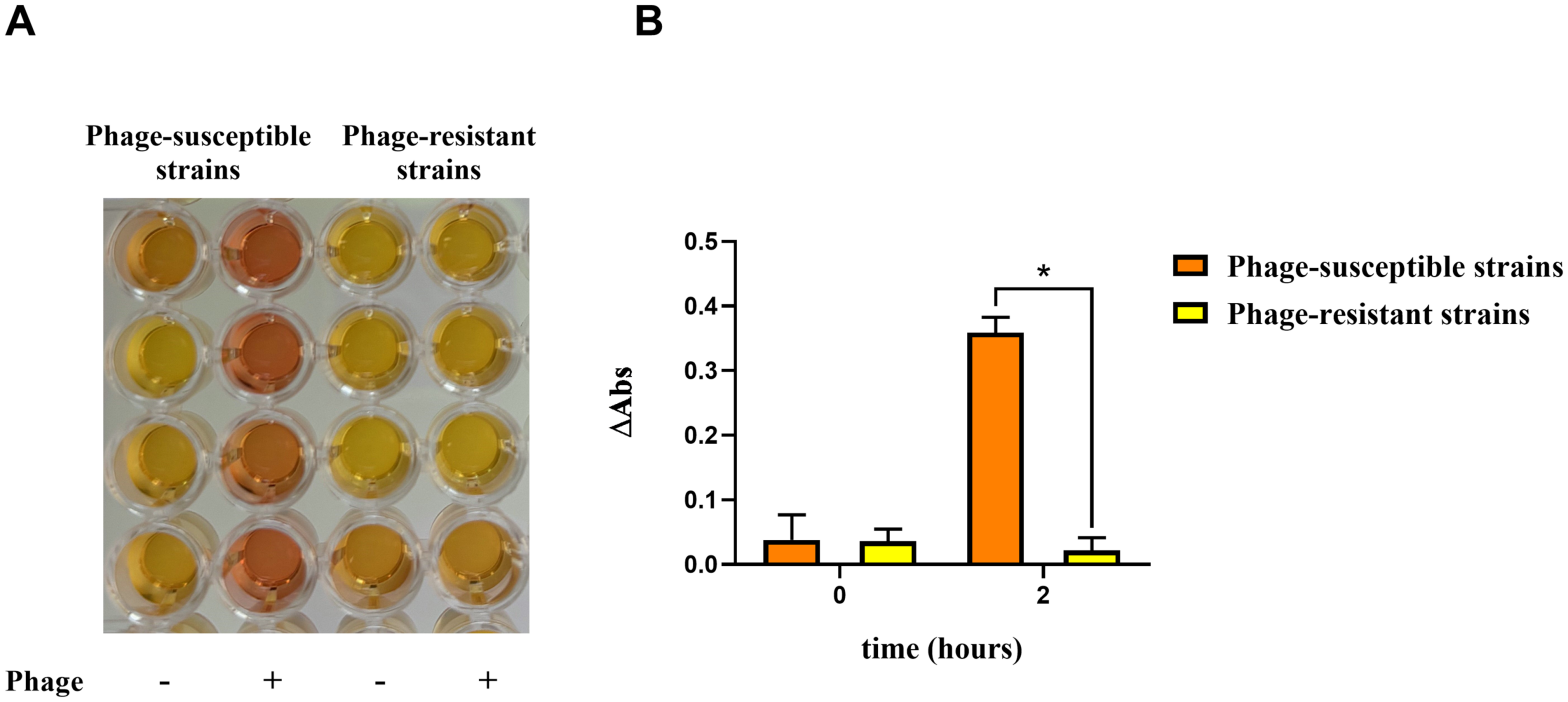
ColorPhAST results. **(A)** Colorimetric test results for 4 phage-susceptible and 4 phage-resistant isolates. **(B)** Increase in absorbance (∆Abs) between 3 phage-susceptible and 3 phage-resistant isolates. *Graph created with GraphPad*. Three independent replicates were performed. The data are means and standard deviations (SD). * *p* ≤ 0.0001.

On the other hand, if the well containing the phages has not changed color, it indicates that the bacteria have not grown because the phage is lysing it. We are therefore dealing with a phage-susceptible isolate (Figures 1B,C and 2A). Noteworthy, a control with test solution and phages was included to rule out possible contamination in both the medium and the phage suspension. In this way, the test is considered invalid when the control turns to yellow or the first well (without phage) of the isolate tested remains orange.

In order to facilitate the automation of the technique, the acidification of the medium was also monitored by measuring absorbance at 569 nm, which corresponds to the optimal wavelength for detecting the red phenol color shift from orange to yellow. Readings were taken at time 0 and after 2 h of incubation at 37°C with shaking. The increase in absorbance (ΔAbs) was calculated as the difference in absorbance between wells with and without phage after incubation (ΔAbs = Abs^phage+^ − Abs^phage-^). At time 0, no significant difference in ΔAbs is observed between phage-resistant and phage-susceptible strains. However, after 2 h of incubation, a clear divergence becomes evident: resistant strains maintain similar absorbance values in both wells, while susceptible strains exhibit a difference in absorbance. This is due to the reduced metabolic activity in the well containing the phage, as bacterial lysis prevents growth (Figure 2B). Importantly, samples must be centrifuged prior to spectrophotometric measurement after 2-h incubation. This step guarantees that the increase in absorbance observed is exclusively due to the pH shift, rather than bacterial growth during incubation.

### 2.7 Statistical tests

The specificity (proportion of phage susceptible isolates that are correctly determined) and the sensitivity (proportion of phage resistant isolates that are correctly determined) were calculated with the ColorPhAST results of 100 *E. coli* clinical strains and using the double agar overlay spot assay as the gold standard method. Moreover, 95% confidence intervals (CI), predictive positive value (PPV), and predictive negative value (PNV) were also estimated (Table 1). True positive results were considered when a clear lysis plaque was observed in the double agar overlay spot assay, while the absence of a clear lysis plaque was considered true negative results.

**Table 1:** Evaluation of ColorPhAST for detecting phage-susceptibility in *E. coli* isolates.

No. of isolates	Double agar overlay spot assay		ColorPhAST		% Sensitivity (95% CI)	% Specificity (95% CI)	% PPV	% NPV
	Positive	Negative	Positive	Negative				
100	45	55	43	57	95.6 (85.2–98.8)	100 (93.5–100)	100	96.5

PPV, positive predictive value; NPV, negative predictive value; CI, confidence interval.

## 3 Results

A total of 100 *E. coli* clinical isolates were tested to evaluate the performance of the ColorPhAST. Previously, all isolates were tested against the phage using the gold standard technique, the double agar overlay spot assay. Within the collection of isolates tested by the gold standard assay, 45 were positive (phage-susceptible) and 55 were negative (phage-resistant). Among the phage-susceptible isolates, 43 isolates were correctly identified as susceptible with the ColorPhAST. However, 2 phage-susceptible isolates gave negative results (phage resistance) with the ColorPhAST. On the other hand, all the phage-resistant isolates had a negative result (phage resistance) with the ColorPhAST. Thus, the evaluation step showed a strong correlation between the ColorPhAST and the gold standard method. The sensitivity, specificity, positive predictive value (PPV), and negative predictive value (NPV) were found to be 95.6, 100, 100, and 96.5%, respectively (Table 1).

Although the rapid test is designed for visual diagnosis (Figure 2A), the possibility of automating the technique was considered; therefore, absorbance at 569 nm was measured for 3 phage-resistant and 3 phage-susceptible strains using a plate reader. Then, the increase in absorbance (ΔAbs) was calculated, considering the ΔAbs as the difference in absorbance between the wells with and without phage (Figure 2B). As shown in Figure 2B, ΔAbs value was similar between the phage-susceptible and resistant strains at time 0, as expected. Nevertheless, ΔAbs was significantly higher in the phage-susceptible strains than that observed in phage-resistant strains after 2 h of incubation. In this way, ΔAbs would indicate the activity of the phage against the strain after 2 h of incubation.

## 4 Discussion

In this study, we developed a rapid colorimetric assay, named ColorPhAST (Color Phage Activity Susceptibility Test), for detecting phage-susceptibility in *E. coli*. This test represents the arrival of a low-cost and rapid diagnostic method that does not require specialized equipment. The cost to test the susceptibility of a bacterial isolates against 94 potential phages, is only 4.5 euros including reagents and 96-well plate. Furthermore, it is a visual colorimetric assay that eliminates the need for specialized technical personnel, offering results that can be observed without the aid of instruments. As we have demonstrated, this technique could also be automated, since ΔAbs after 2 h would indicate susceptibility or resistance. Thus, within just 2 h, a clear and straightforward diagnosis can be made regarding the susceptibility or resistance of the bacterial strain of interest to multiple phages simultaneously (Figure 1B). Nonetheless, further experiments will be required to assess the minimum ΔAbs value necessary to consider an isolate as susceptible to the phage after 2 h of incubation.

As mentioned above, there are several techniques for diagnosing phage susceptibility. Plaque-based assays represent the most traditional and commonly employed methods. This approach includes double agar overlay of phage-bacteria or spot assay as gold standards (Figure 1A), and cross streak phage assay and real-time assessment of plaque formation (Yerushalmy et al., 2023). Those techniques are non-laborious, not expensive, and able to identify phage’s lytic activity. However, it takes 24 h to obtain the results (Glonti and Pirnay, 2022). In contrast, real-time plaque assay can provide results in just 4 h; however, it requires specialized equipment that may not be available in all hospitals settings (Perlemoine et al., 2021).

On the other hand, among the LMT, the optical density assay which monitors bacterial growth as a measure of phage activity is the most used at present. Nevertheless, this method needs a plate reader, the results of which can be affected by the presence of aggregates. Other drawbacks are the slowness of the test (it takes between 24 and 48 h to obtain a reliable result) and the inability to differentiate between live and dead cells (Yerushalmy et al., 2023; Rajnovic et al., 2019). The second LMT is a growth kinetic assay named OmniLog system. It is based on evaluating cell respiration using tetrazolium for indicating bacterial growth by color change. However, this technique does not detect delayed bacterial lysis after growth and requires special equipment and 24–48 h to provide a result (Henry et al., 2012). The third LMT involves the rapid detection (30 min) of phage DNA via fluorescent tagging. Nevertheless, it does not provide identification of inhibition patterns and requires expensive equipment, which is often not available in clinical laboratories (Low et al., 2020).

There are several less common methods for assessing bacteriophage activity, including quantifying the number of surviving bacteria post-phage exposure and surface plasmon resonance imaging (SPRi) (O’Connell et al., 2022). The latter technique, which is highly dependent on specialized equipment, enables the detection of receptor-binding events but does not provide information on the lytic profile. Consequently, it must be used in conjunction with other methodologies to obtain a comprehensive evaluation. Additional approaches for detecting bacterial lysis rely on the secretion of bacterial components, the use of engineered labelled phages (Zelcbuch et al., 2021), microcalorimetry (Tkhilaishvili et al., 2018), quantitative PCR (qPCR) (Liu et al., 2014), and even artificial intelligence-driven analyses (Lood et al., 2022). However, many of these techniques require sophisticated and expensive instrumentation, advanced technical expertise, and labour-intensive sample preparation or data processing, which constrain their broader applicability (Zelcbuch et al., 2021; Tkhilaishvili et al., 2018; Liu et al., 2014; Lood et al., 2022).

Of note, ColorPhAST is very sensitive and specific and has high PPV and NPV (Table 1), being able to detect all the phage-resistant *E. coli* isolates analysed in this study (0% false positives). Only 2 phage-susceptible *E. coli* isolates were not detected by the ColorPhAST (4.4% false negatives). This means that ColorPhAST will not incorrectly identify a bacterium as susceptible if the phage does not target it, thereby preventing false positives and avoiding the possible administration of ineffective phage therapy in patients. Nonetheless, due to its simplicity, the test has some limitations. These include the inability to determine the phage titer, detect late bacterial lysis after growth or partial lysis due to heteroresistance in bacterial population. Moreover, it is not able to distinguish between dead or dormant/inhibited cells, nor identify inhibition patterns.

## 5 Conclusion

ColorPhAST is a rapid and easy-to-perform test, combining excellent sensitivity and specificity. The use of this method is especially interesting in a context of increased prevalence of antimicrobial resistance (AMR) that seems to be higher year by year (Ho et al., 2025). ColorPhAST could play a key role in microbiological diagnostics and personalized phage therapy by allowing faster detection of phage susceptibility, improving the design of phage cocktails.

## Data Availability

The original contributions presented in the study are included in the article/supplementary material, further inquiries can be directed to the corresponding authors.
